# Determining COVID-19's impact on an academic medical library's literature search service

**DOI:** 10.5195/jmla.2022.1447

**Published:** 2022-07-01

**Authors:** Courtney Wombles, Kelsey Grabeel, David Petersen

**Affiliations:** 1 courtney.wombles@lmunet.edu, MSIS, AHIP, Medical Librarian, Lincoln Memorial University DeBusk College of Osteopathic Medicine, Knoxville, TN.; 2 kgrabeel@utmck.edu, Assistant Director of the Health Information Center, Preston Medical Library, Knoxville, TN.; 3 dpetersen@utmck.edu, Research and Learning Services Librarian, Preston Medical Library, Knoxville, TN

**Keywords:** COVID-19 pandemic, health science libraries, literature searching, library services

## Abstract

**Objective:**

At many institutions, literature search services are an important aspect of health science librarianship. This exploratory study analyzes how the COVID-19 pandemic impacted the use of an academic hospital medical library's literature search service.

**Methods:**

To evaluate the pandemic's impact on literature searching at The University of Tennessee Medical Center's Preston Medical Library, data were analyzed for changes from the year before the pandemic (March 1, 2019 to February 29, 2020) to the first year during the pandemic (March 1st, 2020 to February 28, 2021). This was accomplished using LibWizard, a library feedback and assessment application, to review literature search data during the two periods. Variables of interest included total searches, purpose of searches, affiliation of the searcher, and searches with a pandemic-related research question.

**Results:**

A 36.6% drop in literature search service usage was reported from the pre-pandemic year to the during-pandemic year. There was a 55.3% decrease in searches intended for research, as well as significant decreases in the number of searches requested by all patron affiliations. After March 2020, 10% of all searches concerned a COVID-related topic.

**Conclusion:**

The overall decrease in literature search requests, decrease in research searches, decrease in searches among all patron affiliations, and increase in searches on a COVID-related topic suggest that healthcare worker and institutional priorities changed during the pandemic. The results revealed research interests during the first year of the pandemic, as well as an overall change in library service functionality.

## INTRODUCTION

Library literature search services, which provide healthcare workers with ready access to evidence-based resources, demonstrate the library's value by directly impacting patient care [1–3]. Research has examined the literature search service's impact on faculty, residents, and the library itself. Typically, this research falls into several categories: articles discussing the effectiveness of literature search training for faculty and medical residents [4]; articles examining whether a clinical librarian, and by default a literature search service, improves patient outcomes [5]; articles exploring the effectiveness of library resources and literature search service on faculty and resident scholarly activity [6]; and articles that explore the literature search service statistics and data [7].

Literature search service policies can vary depending on the library's institution and staffing. For example, one library specifies how it will share results, the expected time until results, what type of search will be performed based on the search request, and who can request a search [8]. Another biomedical library indicated that the searching librarian would classify the search in one of three tiers, depending on the nature of the search. The tier determines whether the librarian must be included as a coauthor or given acknowledgment [9].

With the onset of the COVID-19 pandemic in 2020, the implementation and utilization of library services changed dramatically in many libraries [10–12]. These changing services created many challenges during the COVID-19 pandemic, such as: handling and processing the vast quantities of COVID-19 literature being produced [13–14], changing library orientations and other classes to a virtual format [15], increasing collaboration with clinicians and departments [16–17], and updating library services to ensure continuity, functionality, and the ability to thrive in a near-virtual environment [10–12, 18–20]. Ayeni et al. conducted a systematic review of library services and challenges caused by the pandemic, finding that libraries were expanding their electronic resources and involvement in virtual education [21]. Other studies suggest that many of these revamped virtual services may be more effective than the previous services offered and might remain even after COVID-19 is no longer a significant threat and many in-person services return [11–12].

Preston Medical Library (PML), located inside The University of Tennessee Medical Center, offers a literature search service to the Graduate School of Medicine (GSM) and University Health Systems (UHS) community. This service provides patrons with a thorough, systemic search of literature on a topic of interest. Those who request a literature search are faculty, residents, staff, nurses, administrators, medical students, and physicians. Requestors state the purpose of their search when requesting. Patrons may request literature for the purpose of patient care, research, a lecture or presentation, or metrics. Only one purpose may be selected per request. Completed search results are typically sent through email; however, some requestors desire printed results. Turnaround time per request can vary depending on the search's complexity and whether the search is requested for patient care. The typical turn-around time is 1 to 2 weeks, as noted on the search request form [22]. Request information is kept in an internal database with data that include name, phone number, email, affiliation, department, purpose, date needed, research question, age, restrictions, special instructions, the librarian who completed the search, search count, and date completed. There is no formal follow-up interview, but requestors are encouraged to contact the librarian conducting the search if they have questions or need different search results. In recent years, data have shown that the library's literature search service has been well utilized, with 582 searches in 2016, 770 searches in 2017, and 571 searches in 2018 [23].

PML closed in-person services from March – June 2020 due to the issuance of Tennessee's Safer at Home Order; however, library staff continued to provide virtual services, including the literature search service [24]. To examine the impact of COVID-19 on the information-seeking behavior of healthcare workers, researchers conducted an exploratory study using data from 2019 and 2020 about the literature search usage and behavior. Researchers theorized that overall usage and searches conducted for research purposes would decrease. However, it was also theorized that the number of patient care literature search requests would increase because of the unknown of the COVID-19 pandemic and the lack of experience in treating the virus.

## METHODS

To assess the COVID-19 pandemic's effect on PML's literature search service, researchers used historic data stored in LibWizard, an application that is part of the LibApps Springshare platform [25]. As library patrons request literature searches,, items such as the requestor's affiliation and department, the purpose of the search, and the research question are documented using a LibWizard form. These may then be retrieved and assessed by generating LibWizard reports on the PML Literature Search Request Form.

Before retrieving the necessary data from LibWizard, researchers determined which search factors should be evaluated. These items were chosen due to the study's exploratory nature and researcher theories that overall searches and research searches would decrease, while patient care searches would increase. Based on this, the following search factors were assessed: the total number of searches performed, the purpose of the searches, and the number of searches on a COVID-related topic. The service's data were analyzed for changes from the year before the pandemic began (March 1, 2019 toFebruary 29, 2020) to the first year during the pandemic (March 1, 2020 to February 28, 2021). March was chosen as the defining month because it was during March 2020 that the World Health Organization (WHO) declared COVID-19 a pandemic and Tennessee's “Safer at Home” Order was issued [24].

Researchers then ran reports in LibWizard to retrieve data. First, the total number of searches for both the pre-pandemic and during-pandemic time frames were retrieved. The submission date advanced filter was first adjusted to pull searches submitted from March 1, 2019, to February 29, 2020. The same was then performed for submission dates from March 1, 2020, to February 28, 2021. Researchers also reported the total number of searches for each month during the pre-pandemic and during-pandemic years.

Next, a field analysis report was run to determine the number of searches with the purposes of research and patient care. Search requests classified as “research” were those intended to be used for hospital studies, personal research interests, scholarly articles, and related projects. Search requests classified as “patient care” were those intended to be used when diagnosing, treating, and/or educating patients. A report was run to retrieve the total number of research searches during the pre-pandemic and during-pandemic periods. The total number of searches for each month during the years was also calculated. The same was then performed for patient care searches.

Following this, a report was run that retrieved all searches whose topic explored an aspect of COVID-19. To achieve this, researchers retrieved all literature searches that contained one or more COVID-related terms within their research question. These terms included “COVID-19,” “pandemic,” and “coronavirus.” The total number of searches containing one or more of these terms was retrieved for both the pre-pandemic and during-pandemic year.

Finally, researchers retrieved data for various patron affiliations. As previously stated, patrons with many different affiliations use PML's literature search service. These include medical students; faculty, staff, and administration; physicians; nurses; and residents and fellows. The total number of searches requested by each affiliation was retrieved for both the pre-pandemic and during-pandemic years.

The field analysis was completed, and researchers then used statistical methods to interpret the results. First, the percentage of total searches intended for research, for the purpose of patient care, concerning a COVID-related topic, and requested by each patron affiliation were calculated for each year. Next, the percentage increases and percentage decreases from year one to year two were calculated for the total number of searches, searches for research, searches for patient care, COVID-related searches, and searches by patron affiliation. Finally, paired, two sample t-tests were conducted to determine the data's statistical significance.

## RESULTS

Reports run within the LibWizard database indicated that the total number of searches from March 1, 2019, to February 29, 2020, was 475, while only 301 searches were completed from March 1, 2020, to February 28, 2021. This revealed a 36.6% decrease in total searches from the pre-pandemic year to the during-pandemic year. A paired, two-sample t-test was conducted to compare the total number of searches during each year. There was a significant difference in the total number of searches for March 1, 2019 toFebruary 29, 2020 (M=39.58, SD=7.61) and March 1, 2020 to February 28, 2021 (M=25.08, SD=9.70); t(11) =4.4413, p = 0.0010, 95% CI [7.31, 21.69]. These results suggested that the onset of the COVID-19 pandemic negatively impacted the total number of literature searches requested by library patrons.

For both the pre-pandemic and pandemic years, the main purpose for using the literature search service was for research, including hospital studies, personal research interests, scholarly articles, and related projects. However, the number of searches intended for research dropped from 313 pre-COVID to 140 during COVID. This indicated a 55.3% decrease in searching used for research and publishing purposes among library patrons. Likewise, the percentage of total literature search requests meant for research dropped from 65.9% to 46.5%. A paired, two sample t-test was conducted to compare the number of research searches during each year. There was a significant difference in the number of research searches for March 1, 2019-February 29, 2020 (M=25.25, SD=7.01) and March 1, 2020-February 28, 2021 (M=11.33, SD=4.83); t(11) =7.4637, p < 0.0001, 95% CI [9.81, 18.02]. These results suggested that the onset of the COVID-19 pandemic had a negative impact on the number of searches intended for research.

**Figure 1:**
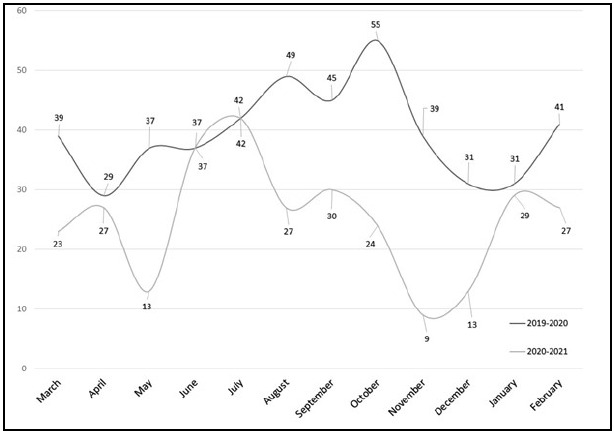
Monthly distribution of total searches submitted during the pre-pandemic year (March 1, 2019-February 29, 2020) and during-pandemic year (March 1, 2020-February 28, 2021).

**Figure 2:**
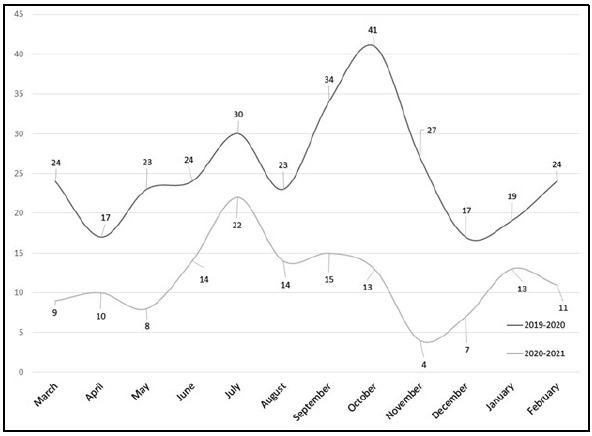
Monthly distribution of searches with the purpose of research during the pre-pandemic year (March 1, 2019-February 29, 2020) and during-pandemic year (March 1, 2020-February 28, 2021).

While the number of searches meant to benefit patient care also dropped from 111 pre-pandemic to 80 during the pandemic (a 27.9% decrease), the percentage of total searches meant for patient care rose from 23.3% to 26.6% during the pandemic. A paired, two sample t-test was conducted to compare the number of patient care searches during each year. There was not a significant difference in the number of patient care searches for March 1, 2019-February 29, 2020 (M=9.25, SD=1.86) and March 1, 2020-February 28, 2021 (M=6.67, SD=4.87) conditions; t(11) =1.7901, p = 0.1010, 95% CI [-0.59, 5.76]. These results suggested that the onset of the COVID-19 pandemic did not have a positive impact on the number of searches intended for patient care.

**Figure 3:**
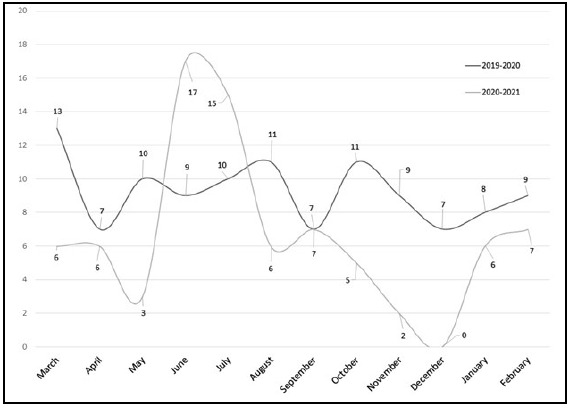
Monthly distribution of searches with the purpose of patient care during the pre-pandemic year (March 1, 2019-February 29, 2020) and during-pandemic year (March 1, 2020-February 28, 2021).

**Figure 4:**
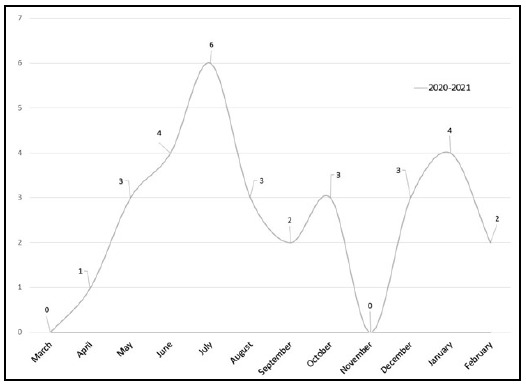
Monthly distribution of searches including “COVID” in their research question from March 1, 2020, to February 28, 2021.

There were no searches before March 2020 that concerned a COVID-related topic. However, 10% of all searches, a total of 30, made from March 1, 2020, to February 28, 2021, included pandemic-related terms. Each of the 30 searches included the term “COVID-19” in its research question. Neither “pandemic” nor “coronavirus” appeared in any searches. The monthly frequency of these searches is reported in Figure 4.

In the past, the patrons who most frequently utilized PML's literature search service were nurses, and this remained true during the first year of the pandemic. However, the number of nurses who requested literature searches dropped from 187 (39.4% of all searches) in the pre-pandemic year to 86 (28.6% of all searches) in the during-pandemic year. This indicated a 54% decrease in literature searches requested by nurses after COVID-19 was declared a pandemic. All other affiliations also showed decreases in literature search service use after March 1, 2020. A one-way repeated measures ANOVA was performed to compare the effect of the COVID-19 pandemic on literature searches requested by different patron affiliations. The ANOVA revealed that the onset of the pandemic lead to statistically significant differences in the number of literature search requests (F (1, 7) = [7.13], p = [0.03]).

**Figure 5:**
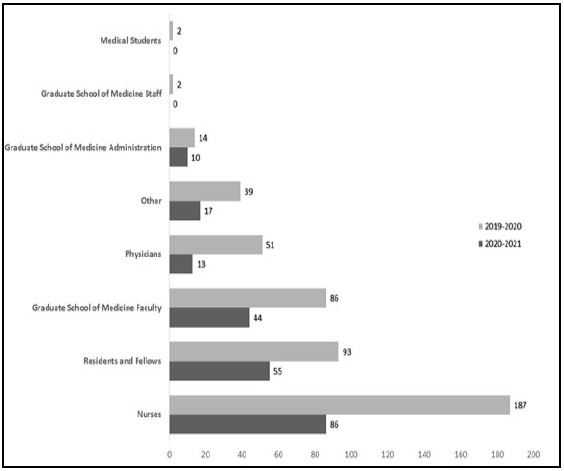
Distribution of searcher affiliations during the pre-pandemic year (March 1, 2019-February 29, 2020) and during-pandemic year (March 1, 2020-February 28, 2021).

## DISCUSSION

By analyzing changes in LibWizard data from the year before the pandemic began (March 1, 2019 to February 29, 2020) to the first year during the pandemic (March 1, 2020 toFebruary 28, 2021), it was determined that the onset of the COVID-19 pandemic had a significant impact on PML's literature search usage and behavior.

First, this study found a significant decrease in the total number of literature searches requested by library patrons after the pandemic's onset. This study also revealed that the onset of the COVID-19 pandemic had a negative impact on the number of searches for all patron affiliations. Researchers have theorized that the drop in literature searches was partially due to PML's closure to in-person services at the beginning of the pandemic. Patrons frequently request literature searches by visiting the library, and it is believed that removing this option, as well as uncertainty surrounding available services during closure, may have caused decreased usage. The drop in literature search service usage may have also resulted from the COVID-19 surge of inpatients. In Tennessee, COVID-19 was first reported on March 5, 2020, and then spread throughout the state [26]. By the end of 2020, statewide COVID-19 hospitalization numbers had reached 3,077 (floor and Intensive Care Unit (ICU)), impacting every region [27]. The number of COVID-positive inpatients in all Knox County hospitals rapidly changed from 0 on March 1, 2020, to a high point during this study period of 671 inpatients on January 7, 2021 [28]. Healthcare providers may have been too occupied by patient care to utilize library services; physical and mental exhaustion among healthcare professionals was widely reported, as well as changes in the delivery of healthcare [29]. As the pandemic progressed and COVID cases increased, so did the workload of healthcare providers [30]. As healthcare professionals adjusted to these changes and dealt with an unprecedented hospital environment, use of the literature search service may have declined in concert with their changing workflows.

Second, this study found a significant decrease in the number of literature searches intended for research after March 2020. Prior to the pandemic, patrons' main purpose for requesting searches was for research, which included hospital studies, personal research interests, scholarly article publication, and related projects. After the pandemic's onset, however, research searches underwent a 55.3% decrease. Researchers have theorized that the decrease in searches used for research purposes might have been due to the cancellation of conferences because of the pandemic, as well as an increase in patient care responsibilities. Furthermore, healthcare providers might have lost their dedicated research time with the increase in inpatients. This may be supported by existing literature that links higher burdens on the healthcare system to reduced opportunities for medical publishing, particularly during the COVID-19 pandemic [31].

Not only does this study reveal changes in a library's literature search service, but it also allows reflection on how the practices of health science librarians may change going forward. By noting how library services and functionality were altered by the onset of the COVID-19 pandemic, best practices for future public health crises may be determined. Just as the healthcare professionals served by the library must change their priorities and workflows during challenging times, so must the library itself. For example, the findings of this exploratory study suggest that library services should be prepared to serve users in an online environment, asserting that their services are still available. Health science librarians should be prepared to address an increase in questions about the topic of the health crisis itself, as well as combat misinformation surrounding it. A decline in usage of library research support services can be expected, as patron priorities shift more toward clinical work and direct patient care. Librarians should pivot to support this clinical work, recognizing the physical and mental exhaustion among health care professionals and providing literature search services that keep patrons up to date on the public health crisis and directly support the treatment of patients.

In conclusion, this study examined the impact of COVID-19 on PML's literature search service, adding to existing literature that explores search service statistics and data. The results of this study helped to classify the nature of scholarly activity during the pandemic, especially among a health science library and its healthcare provider patrons. The results also revealed how research activity and library services were affected by the pandemic by comparing their outputs during the years preceding and following the onset of COVID-19. Although this study only analyzed one health science library's literature search activity, the methods and scope could be expanded to assess the COVID-19 pandemic's effect on literature searching at other health science libraries, within other library sectors, or within other services provided by libraries. Future research might be needed to determine if the literature search requests used for research led to publications during the pandemic. Additionally, further analysis is needed to determine if the patient care searches had any impact on patient care practices. Researchers plan to review the data in the years following the pandemic to analyze and better understand search behavior.

## Data Availability

Data associated with this article are available in the Open Science Framework https://osf.io/cyrkm/?view_only=e7da454f7844460d83a841ff997b667d

## SUPPLEMENTAL FILES

**Appendix A:** Literature Search Request LibWizard Form
