# Supplementary material for: Determining COVID-19's impact on an academic medical library's literature search service
Source: J Med Libr Assoc. 2022 Jul 1;110(3):316–22. doi: 10.5195/jmla.2022.1447 (PMC9782504; doi:10.5195/jmla.2022.1447)
Supplement: Supplementary file 1 — Appendix A: Literature Search Request LibWizard Form [file jmla-110-3-316-s01.pdf]

Preston Medical Library Literature Search Request

Full Name (required)

Phone

(999)999-9999

Email (required)

Affiliation (required)

Make a selection

Department

Make a selection

Purpose - Please use the category that fits best (required)

Make a selection

Date Needed - If there's a specific date you need the results by, please enter here. **For rush searches needed in 48 hours or less,** PLEASE CALL 865-305-9525. If you do not have a specific time frame, typical turnaround time is 1-2 weeks.

MM/DD/YYYY

Please describe your question:
What would you like to know? What do you already know about the topic?
What will you use this information for?

If applicable to your question, please use the PICO framework: Patient, Intervention, Comparison, Outcome

Patient Type or Problem: How would you describe a group of patients similar to yours? What are the most important characteristics of the patient, population, or problem?

Intervention: What main intervention are you considering?

Comparison (optional): What is the main alternative being considered, if any?

Outcome/Intended Result: What are you trying to accomplish, measure, improve or affect?

Limit by Age

- ◯ Adults
- ◯ Children
- ◯ Either or N/A
- ◯ Specific age group:

Date Restrictions

Make a selection

Special Instructions: method of delivery other than email (such as in an EndNote library), other search limits, or details.

Library Use Only: already assigned/completed? (required)

No

Submit
